# Extracorporeal shock waves alone or combined with raloxifene promote bone formation and suppress resorption in ovariectomized rats

**DOI:** 10.1371/journal.pone.0171276

**Published:** 2017-02-03

**Authors:** Adriano Lama, Anna Santoro, Bruno Corrado, Claudio Pirozzi, Orlando Paciello, Teresa Bruna Pagano, Sergio Russo, Antonio Calignano, Giuseppina Mattace Raso, Rosaria Meli

**Affiliations:** 1 Department of Pharmacy, University of Naples Federico II, Naples, Italy; 2 Program in Integrative Cell Signaling and Neurobiology of Metabolism, Department of Obstetrics, Gynecology, and Reproductive Sciences, Yale University School of Medicine, New Haven, Connecticut, United States of America; 3 Department of Public Health, School of Medicine and Surgery, University of Naples Federico II, Naples, Italy; 4 Department of Veterinary Medicine and Animal Production, University of Naples Federico II, Naples, Italy; China Medical University, TAIWAN

## Abstract

Osteoporosis is a metabolic skeletal disease characterized by an imbalance between osteoclast-mediated bone resorption and osteoblast-mediated bone formation. We examined the beneficial effect of shock waves (SW) alone or in combination with raloxifene (RAL) on bone loss in ovariectomized rats (OVX). Sixteen weeks after surgery, OVX were treated for five weeks with SW at the antero-lateral side of the right hind leg, one session weekly, at 3 Hz (EFD of 0.33 mJ/mm^2^), or with RAL (5 mg/kg/die, per os) or with SW+RAL. Sera, femurs, tibiae and vertebrae were sampled for following biochemical and histological analysis. SW, alone or combined with RAL, prevented femur weight reduction and the deterioration of trabecular microarchitecture both in femur and vertebrae. All treatments increased Speed of Sound (SoS) values, improving bone mineral density, altered by OVX. Serum parameters involved in bone remodeling (alkaline phosphatase, receptor activator of nuclear factor kappa-B ligand, osteoprotegerin) and osteoblast proliferation (PTH), altered by ovariectomy, were restored by SW and RAL alone or in combination. In tibiae, SW+RAL significantly reduced cathepsin k and TNF-α levels, indicating the inhibition of osteoclast activity, while all treatments significantly increased runt-related transcription factor 2 and bone morphogenetic-2 expression, suggesting an increase in osteoblastogenic activity. Finally, in bone marrow from tibiae, SW or RAL reduced PPARγ and adiponectin transcription, indicating a shift of mesenchymal cells toward osteoblastogenesis, without showing a synergistic effect. Our data indicate SW therapy, alone and in combination with raloxifene, as an innovative strategy to limit the hypoestrogenic bone loss, restoring the balance between bone formation and resorption.

## Introduction

Osteoporosis is a metabolic skeletal disease characterized by low bone mass, deterioration of bone micro-architecture and increased fracture risk [1]. The prominent prevalence of osteoporosis in Europe (approximately 21% of women aged 50–84 years) and increased mortality rate in patients with osteoporotic fractures represent a clinical emergency [2, 3]. Postmenopausal women exhibit the major risk to develop osteoporosis, suggesting that the role of estrogen is critical in its pathogenesis [4]. Indeed, raloxifene, a selective estrogen receptor modulator (SERM), has been approved for the prevention and treatment of postmenopausal osteoporosis, especially because of its capability to prevent or reduce vertebral fractures [5]. However, the efficacy of this drug in reducing or preventing non-vertebral fractures is strongly limited [6].

The osteoporosis progression is due to an imbalance between osteoclast-mediated bone resorption and osteoblast-mediated bone formation. The recovery of this balance represents the rationale underlying the two more recent two anti-osteoporotic strategies: the inhibition of bone resorption and turnover, and the stimulation of bone formation [7, 8]. Cathepsin k, a protease abundantly expressed in osteoclasts and in actively resorbing osteoclasts, has been identified as a novel pharmacological target to counteract osteoporosis by reducing the organic bone matrix degradation. In particular, two cathepsin k inhibitors, odanacatib and ONO-5334, have been recently used in clinical trials [9, 10].

To date, the only available agents that stimulate bone formation are the whole molecule parathormone (PTH, 1–84) or its fragment, the teriparatide (1–34). Although PTH increases bone formation through an increase in bone remodeling, its effect is transient and decreases with time [11, 12]. Moreover, the use of PTH analogues in the clinical practice is limited by their cost and potential side effects [13].

In the early 80s, shock waves (SW) were used for kidney and urinary stone lithotripsy [14]. Afterwards, SW therapy has been used for the treatment of other orthopedic diseases, accelerating bone healing [15], callus formation [16] and delayed or non-union of long bone fractures [17]. In addition, SW have been shown to promote the regeneration of alveolar bone in a rodent model of periodontitis [18]. This therapy is considered a safe and highly versatile tool to enhance the time of tissue regeneration, in particular on tendon and muscle tissues, also showing immediate antalgic and anti-inflammatory effects [19].

The biological effects of the SW therapy in bone have been recently examined [20]. Indeed, Van der Jagt et al. [21] have demonstratedt hat single application of SW has a light beneficial effect in a rat model of ovariectomy-induced osteoporosis, increasing trabecular bone volume and reducing bone loss. Additionally, this research group showed that a single application on tibia induces anabolic effects in cortical bone in normal [22] and osteoporotic rats, especially when SW treatment was combined with anti-resorptive alendronate therapy [23]. However, the bone biochemical mechanisms underlying the anti-osteoporotic effects of SW are still overlooked.

The purpose of this study was to evaluate the modulation of serum parameters and tissue markers of bone resorption and bone formation in ovariectomized rats after repeated SW therapy, alone or in combination with raloxifene.

## Materials and methods

### Animals

Female Sprague Dawley rats (Harlan Italy, San Pietro al Natisone, Udine, Italy) were housed in stainless steel cages in a room kept at 22±1°C with a 12:12 h light-dark cycle. All procedures involving the animals were carried out in accordance with the Institutional Guidelines and complied with the Italian D.L. no.116 of January 27, 1992 of Ministero della Salute and associated guidelines in the European Communities Council Directive of November 24, 1986 (86/609/ECC). All animal procedures reported herein were approved by the Institutional Animal Care and Use Committee (CSV) of University of Naples Federico II under protocol no. 2013/0040366. Prior to sample and tissue collection, animals were euthanized by an intraperitoneal injection of a cocktail of ketamine/xylazine, followed by cervical dislocation to minimize pain. All efforts were made to minimize animal suffering.

### Ovariectomy, Shock Waves (SW) and pharmacological treatment

**Fig 1:**
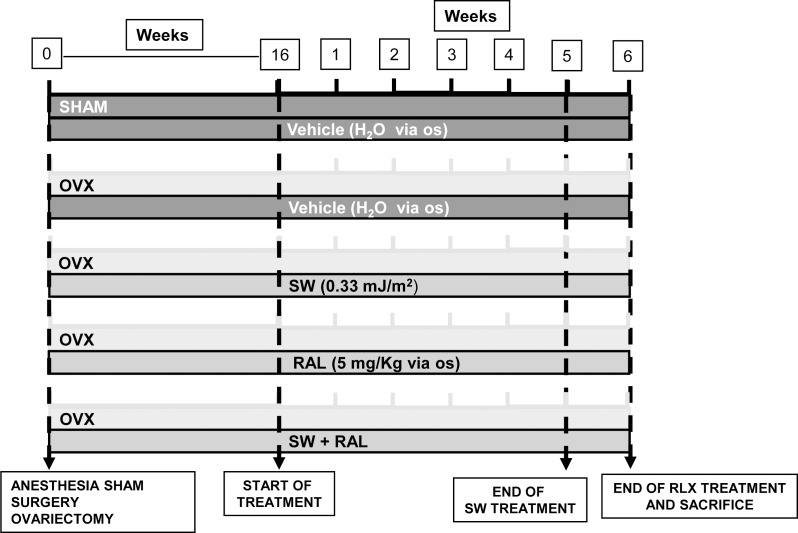
Experimental scheme. At the onset of the study, female rats were bilaterally ovariectomized (OVX) while the sham-operated (SHAM) animals were used as controls. After sixteen weeks, rats were divided into five groups: 1) SHAM, receiving drug vehicle; 2) OVX, receiving drug vehicle; 3) OVX treated with SW once a week for five weeks; 4) OVX, receiving RAL (5 mg/kg/die *per os* for five days a week for six weeks); and 5) OVX rats treated with SW in combination with RAL.

The experimental design is illustrated in Fig 1. At the onset of the study, female rats (mean body weight of the cohort: 216.4 ± 1.6 g) were bilaterally ovariectomized (OVX) under anesthesia (100 mg kg^-1^ ketamine plus 5 mg kg^-1^xylazine ip). The sham-operated (SHAM) animals were subjected to the same general surgical procedure as OVX groups except for ovarian excision. After surgery, SHAM and OVX animals received topical antibiotic treatment (with a preparation containing 0,05 g of neomycin sulfate and 9,95 g of sulfathiazole, for five days).

Sixteen weeks after surgery, necessary time for the development of osteoporotic features [24], rats were divided into the following five groups (n = 6 animals each group): 1) SHAM, control rats receiving *per os* drug vehicle; 2) OVX, rats receiving vehicle; 3) OVX+SW, rats treated with SW one weekly session for five times; 4) OVX+RAL, rats receiving raloxifene (5 mg/kg/die, Lilly Research Laboratories, Indianapolis, USA) *per os* five days a week for six weeks; and 5) OVX+SW+RAL, rats treated with SW in combination with the SERM.

Briefly, every week all rats receiving SW were anesthetized with a mix of oxygen and 2% isoflurane, and the right hind leg was shaved and an ultrasonic gel was applied, as coupling media. The SW applicator was placed at the antero-lateral side of the right thigh and each session of SW treatment consisted in the application of 1000 electro-magnetically generated SW at 3 Hz with an energy flux densities (EFD) of 0.33 mJ/mm^2^ (Duolith^®^ SD1-Storz Medical AG, Tagerwilen, Switzerland). The focus diameter of the Duolith was 4 mm Full Width at Half Maximum (FWHM) at the energy used. Non-SW treated animals were also undergone an anesthesia and shaving without SW application. All animals were euthanized one week after 5th SW application, while last administration of raloxifene was performed 2 h before killing. During necropsy, we confirmed the effectiveness of OVX surgery, through the evaluation of uterus weight. Subsequently, bone tissues (treated and contralateral femurs, tibiae, vertabrae) were sampled and surrounding tissues (skin, muscle and tendons) were removed. Tibiae were immediately frozen in liquid nitrogen for following molecular analyses of bone tissue and bone marrow, whereas femurs and lumbar vertebrae were fixed in 10% Neutral Buffered Formalin (NBF) and subjected to histomorphological examination. Before fixing, femurs were dried to check their weight, distinguishing SW treated bone from SW-untreated ones.

### Body weight gain and fat mass

Throughout the experimental period, body weight was assessed once per week and body weight gain was calculated as difference between the last measure and the body weight recorded at the beginning of the experiment. Lastly, values of body weight gain during time were cumulated and expressed as area under curve (AUC). At the start of necropsy procedure, bioelectrical impedance analysis was used to assess the rat body composition with the BIA 101analyzer, modified for the rat (Akern, Florence, Italy). Fat-free mass was calculated using the bioelectrical impedance analysis (50 kHz) prediction equation of Ilagan et al. [25], and fat mass content was determined as the difference between body weight and fat-free mass.

### Analysis of serum parameters

At the start of necropsy procedure, rats were anesthetized by enflurane and blood was collected by cardiac puncture. Sera were obtained by centrifugation at 1500 x g at 4°C for 15 min, and stored at -70°C for later biochemical and hormonal determinations. Osteoprotegerin (OPG), Receptor activator of nuclear factor kappa-B ligand (RANKL), and alkaline phosphatase (ALP) were measured by Enzyme-Linked ImmunoSorbent Assay (ELISA) kits for rats purchased from Cloud Clone Corp. (Houston, TX, USA), while rat intact PTH (iPHT) levels were determined by an ELISA kit obtained from MyBioSource Inc. (San Diego, CA, USA). All procedures were performed following the manufacturer’s protocol.

### Histological analysis and ultrasonometry

Extracted bones (femurs and vertebrae) were fixed for 3 days in 10% neutral buffered formalin. Then, the fixed tissues were decalcified with a 4% trichloroacetic acid solution and embedded in paraffin. Each femur was cut at a 4 micrometer sections through the longitudinal axis, while each vertebra was cut through the transversal axis. Finally, hematoxylin and eosin (H&E) staining was performed for their morphological evaluation. Slides were analyzed using an optic microscope Nikon Eclipse E600. A blind examination of the sections based on primary histological analysis of bone (modified from Dempster et al. [26]) was made independently by two veterinary pathologists (OP and TBP) at high (20X) and low (10X) magnification.

The ultrasound measurements (QUS) were performed using a DBM Sonic 1200 (IGEA, Carpi, Italy) with an electronic high-precision caliper (± 0,02 mm) where two ultrasound probes (diameter: 12 mm) are mounted: one probe generates the ultrasound (1,25MHz) and the other one receives the ultrasound beam after it has crossed the bone specimen. To obtain the values of speed of sound (SoS, m/s), we used an ultrasonic contact gel along the longitudinal axis of animal femurs. After four measurements, the speed of sound (SoS, m/s) was calculated after four measurements in order to reduce repositioning errors, as previously described [27].

### RNA extraction and Real-time semi-quantitative PCR

Tibiae were dissected from the rats and cleaned with 0.1 M ice-cold PBS, pH 7.2. For OVX+SW and OVX+RAL+SW groups, both contralateral and SW-treated tibiae were used. The epiphysis of each bone was excised and the bone marrow was collected [28]. Bone tissues were snap-frozen in liquid nitrogen to make them breakable. Total RNA was extracted using TRIzol Reagent (Bio-Rad Laboratories), according to the manufacturer’s instructions. cDNA was synthesized using a reverse transcription kit (NucleoSpin^®^, MACHEREY-NAGEL GmbH & Co, Düren, Germany) from 2 μg total RNA. PCRs were performed with a Bio-Rad CFX96 Connect Real-time PCR System instrument and software (Bio-Rad Laboratories). The PCR conditions were 15 min at 95°C followed by 40 cycles of two-step PCR denaturation at 94°C for 15 s, annealing at 55°C for 30 s and extension at 72°C for 30 s. Each sample contained 1–100 ng cDNA in 2X QuantiTect SYBRGreen PCR Master Mix and primers, TNF-α, RANKL, OPG, Bone morphogenetic 2 (Bmp2), runt-related transcription factor 2 (RUNX2), cathepsin k, Peroxisome proliferator-activated receptor gamma (PPARγ) and adiponectin (both by Qiagen, Hilden, Germany) in a final volume of 50 μl. The relative amount of each studied mRNA was normalized to GAPDH as housekeeping gene, and data were analyzed according to the 2^-ΔΔCT^ method.

### Statistical analysis

All data were presented as mean ± SEM. Statistical analysis was performed by ANOVA test for multiple comparisons, followed by Bonferroni’s test. Statistical significance was set at P <0.05.

## Results

### Body parameters

**Fig 2:**
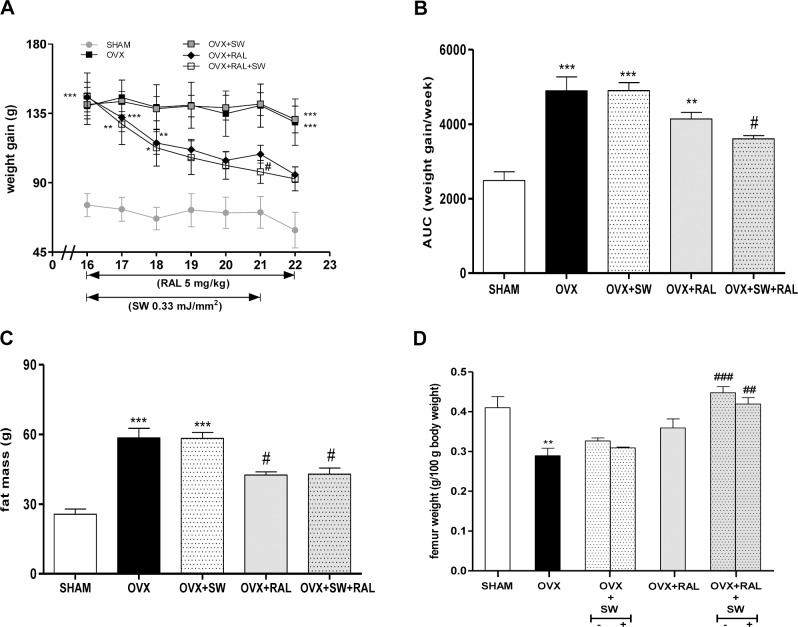
Effects of SW and RAL treatment on body weight gain (A), also expressed as AUC of cumulative weight gain during time (B), fat mass (C) and femur weight (D) are shown. Body weight gain was monitored every week, during all experimental time. Femur weight was determined in contralateral (-) and SW-treated (+) of OVX+SW and OVX+RAL+SW animals. Values are expressed as means ± SEM (n = 6); *P <0.05, **P <0.01 and ***P <0.001 compared with SHAM; #P <0.05, ##P <0.01 and ###P <0.001 compared with OVX.

As depicted in Fig 2A, weight gain of OVX+RAL+SW group decreased during time and was significantly lower than OVX rats after 5 weeks of the combined treatment (21^th^ week). RAL treatment of OVX rats showed a decreasing trend in body weight gain compared to OVX group. Weight gain of OVX+SW did not differ significantly from OVX group. All these data were confirmed by AUC of weight gain during the treatment time (Fig 2B). As shown in Fig 2C, fat mass, measured at 22^th^ week, was significantly increased in the OVX rats, compared with the SHAM animals, while the treatment with RAL or its association with SW reduced it.

The femurs of rats were weighed after excision, discerning the SW treated from untreated bone (Fig 2D). The SW+RAL associated treatment significantly improved the remarkable loss of bone weight of OVX animals.

### Bone histology and serum remodeling markers

**Fig 3:**
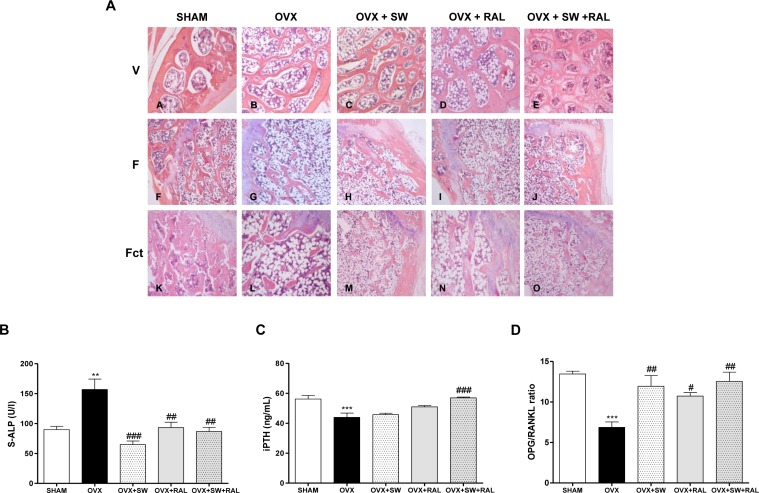
Effects of SW and RAL treatment on histological features of vertebrae (V), femural metaphyseal bone (F) and contralateral femurs (Fct) of SHAM (A,F,K), OVX (B,G,L), OVX+SW (C,H,M), OVX+RAL (D,I,N) and OVX+SW+RAL (E,J,O) groups (panel A). Bone sections were stained with H&E to visualize trabecular thickness and organization, and adipose tissue infiltration in bone marrow. Effects of SW and RAL treatments on bone markers in serum: ALP (panel B), iPTH (panel C) and OPG/RANKL ratio (panel D). Values are expressed as means ± SEM (n = 6); **P <0.01 and ***P <0.001 compared with SHAM; #P <0.05, ##P <0.01 and ###P <0.001 compared with OVX.

We evaluated histomorphology of vertebrae (V) and femurs (F) excised from all rats. Trabecular thickness and organization, and adipose tissue infiltration in bone marrow were examined (Fig 3A). OVX rats showed, as expected, a reduced thickness and organization of vertebra and femur trabeculae, and an increased amount of adipose tissue infiltration compared with SHAM group. In particular in vertebrae, SW and its combination with RAL induced a reduction of adipose tissue infiltration and an improvement of trabecular thickness and organization compared with OVX. The same parameters were also analyzed on femur metaphyses, distinguishing SW treated and contralateral limb (Fct). Thickness and organization of metaphyseal trabeculae were remarkably ameliorated by SW or RAL alone and notably their combination restored trabecular structure as that of SHAM, without difference between SW treated and contralateral limb.

By the quantitative ultrasound measurement, we calculated the SoS of the femur of all animals. OVX rats showed a significant decrease of SoS than SHAM group, improved by all treatments (SHAM, 1931± 6,85; OVX, 1826 ± 4,251 ^***^; OVX+SW, 1867 ± 9,291 ^#^; OVX+RAL, 1887 ± 13,58 ^###^; OVX+SW+RAL, 1864 ± 5,37 ^#^; ^***^ p<0,001 vs SHAM; ^#^ p<0,05 and ^###^ p<0,001 vs OVX).

Serum parameters involved in bone formation or osteoblast proliferation, S-ALP and iPTH respectively, were examined (Fig 3B and 3C). Both markers were modulated by all treatments and in particular SW+RAL was able to significantly restore their levels. Moreover, we showed that treatments with SW, RAL and their combination increased OPG/RANKL ratio showing a reduction of the osteoclastogenic process (Fig 3D).

### SWs alone or combined with RAL modulate genes involved in bone formation and resorption

**Fig 4:**
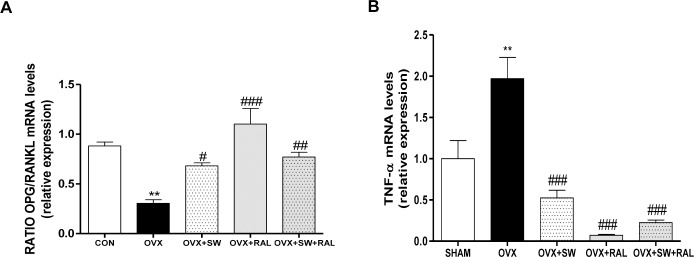
Effect of SW and RAL treatments on bone resorption parameters in tibiae. mRNA of OPG/RANKL ratio (A) and TNF-αare shown (B). For OVX+SW and OVX+RAL+SW groups, contralateral and SW-treated tibiae were used. Values are expressed as means ± SEM (n = 6); **P <0.01 compared with SHAM; #P <0.05, ##P <0.01 and ###P <0.001 compared with OVX.

**Fig 5:**
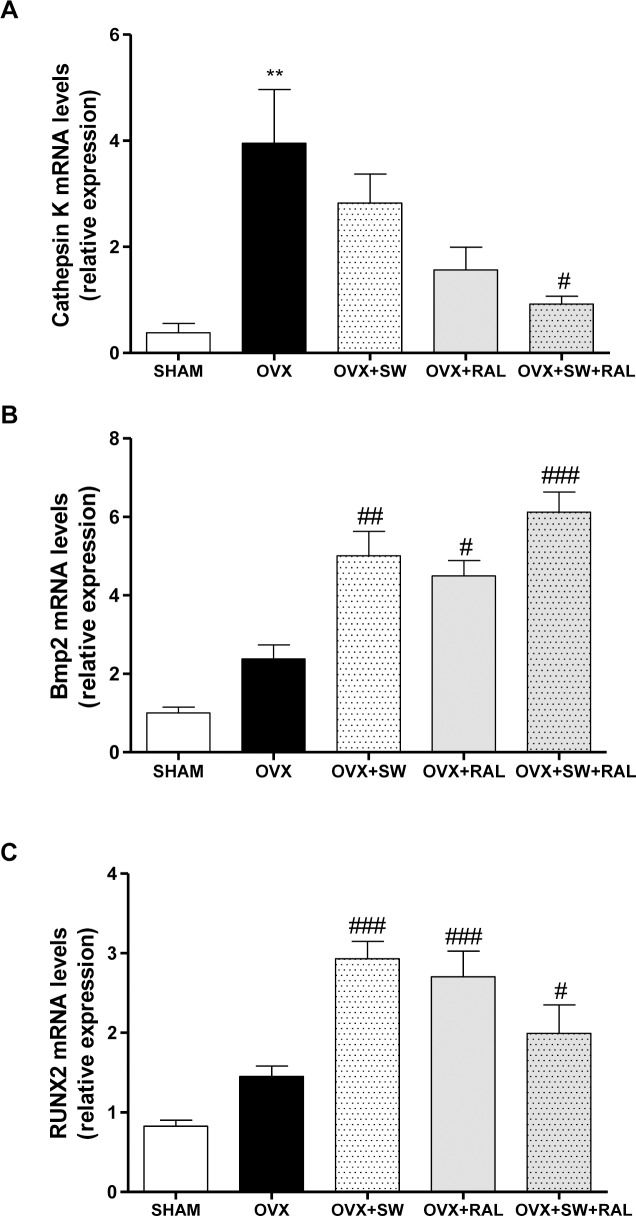
Effects of SW and RAL treatments on bone remodeling balance: cathepsin K (A), Bmp2 (B) and RUNX2 (C) mRNA expression are shown. For OVX+SW and OVX+RAL+SW groups, contralateral and SW-treated tibiae were used Values are expressed as means ± SEM (n = 6); **P <0.01 compared with SHAM; #P <0.05, ##P <0.01 and ###P <0.001 compared with OVX.

To confirm the involvement of OPG/RANKL in the effect of SW and RAL, we also evaluated their mRNA expression by Real Time PCR in tibiae. OVX group showed a significant reduction of OPG/RANKL ratio compared with SHAM, that was significantly increased by SW and RAL, alone or in combination (Fig 4A), indicating a reduction in bone resorption. Consistently, OVX animals showed a significant increase of TNF-α which stimulates osteoclast proliferation, while all treatments remarkably reduced its transcription (Fig 4B). Fig 5 showed the mRNA expression of cathepsin K, Bmp2, andRUNX2. OVX caused a marked increase of cathepsin K compared with SHAM; SW and RAL alone showed a trend of enzyme reduction that reached significance when combined (Fig 5A). Moreover, SW and RAL alone or combined increased Bmp2 mRNA expression(Fig 5B), while SW or RAL alone increased RUNX2 transcription, that was less affected by their combination(Fig 5C).

### Modulation of PPARγ and adiponectin transcription in tibial bone marrow by SW alone or combined with RAL

**Fig 6:**
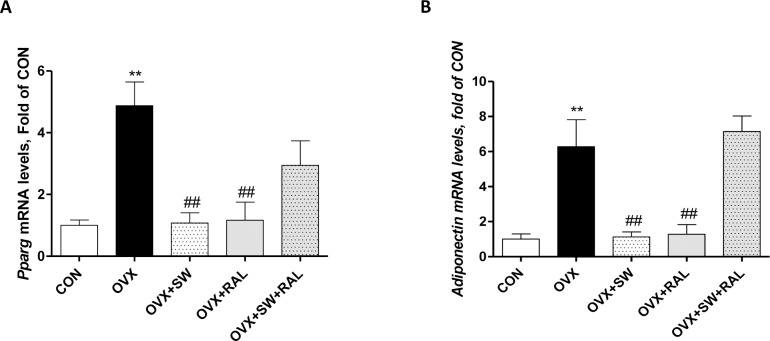
Modulation of PPARγ and adiponectin transcription in tibial bone marrow by SW alone or combined with RAL. PPARγ (A) and adiponectin (B) mRNA expression are shown. For OVX+SW and OVX+RAL+SW groups, contralateral and SW-treated tibiae were used. Values are expressed as means ± SEM (n = 6); **P <0.01 compared with SHAM; ##P <0.01 compared with OVX.

Hypoestrogenism in rodent, as well as in human, was found to be associated with increased bone marrow adiposity [29, 30]. Consistently, we showed an increase in PPARγ and adiponectin mRNAs in bone marrow of tibiae from OVX (Fig 6A and 6B). Both treatments either SW or RAL significantly reduced the transcription of both genes. Conversely, SW and RAL combination did not significantly modify OVX-induced increase in PPARγ and adiponectin mRNAs, although a trend of decrease of PPARγ expression was found.

## Discussion

In the present study we show the beneficial effect of SW therapy, alone or in combination with raloxifene, inducing bone formation and reducing bone resorption impaired by OVX.

Postmenopausal hypoestrogenism triggers the events involved in the osteoporotic process: massive bone resorption entailing loss of bone mass and architecture and failure to replace lost bone due to remarkable reduction of bone formation [4]. The ovariectomized rat is a standard preclinical model in the development of anti-osteoporosis therapies, reproducing molecular and biochemical alterations of the pathology [24, 31].

Our hypothesis regarding the benefits of SW treatment in osteoporosis was suggested by previous data demonstrating that a single SW application could be effective increasing trabecular bone volume and reducing bone loss [21], especially when it was combined with anti-resorptive alendronate therapy [23]. Here, we demonstrate the efficacy of repeated SW applications alone or combined with raloxifene (for 6 weeks) on osteoporosis induced by long term ovariectomy. As previously described [31], and here confirmed, 22 weeks ovariectomy clearly led to a significant increase in body weight and fat mass, and raloxifene reduced fat mass by its estrogenic activity. In our experimental conditions RAL, administered when ovariectomy-induced hypoestrogenism was defined, reduced also significantly weight gain only in association with SW treatment.

Interestingly, when RAL is associated with SW, a synergistic effect is also shown on femur weight since this parameter raised significance only in SW+RAL-treated rats both in SW treated and contralateral femur, indicating a lower bone loss than in OVX animals. In addition, all treatments were able to increase the values of SoS reduced by OVX, indicating an improvement of bone mineral density (BMD).As previously demonstrated, QUS technique is significantly comparable to the dual X-ray absorptiometry (DXA), the main used technology for BMD evaluation [32].

The capability of both treatments in restoring bone architecture was also evidenced by histological analysis. All SW-based treatments were more effective than RAL in reversing histological features of osteoporosis showed in OVX rats, not only in vertebrae but also in treated and contralateral femurs. The evaluation of trabecular architecture in osteoporotic metaphyseal femur shows its considerable improvement after repeated weekly applications of SW. This effect was more evident after combined therapy. We also observed an increase of adipose infiltration in OVX group compared to control and treated groups, not only in femur but also in vertebrae. No differences between treated and contralateral limbs were noticed in SW alone or combined with RAL, supporting the hypothesis of a systemic effect of SW therapy.

Many key factors regulate the balance between bone resorption and formation. Osteoclastogenesis is stimulated by the binding between RANK and RANKL, expressed in osteoclast progenitor cells and osteoblasts, respectively. RANKL-RANK interaction is prevented by the natural RANKL inhibitor, OPG. Clinical and experimental studies demonstrated that estrogens increase mRNA and protein expression of OPG and decrease RANKL and macrophage colony-stimulating factor (M-CSF) expression [33, 34]. In our experimental model, we demonstrate that not only RAL, but also SW and their association inhibit osteoclastogenesis, modulating OPG/RANKL ratio both in serum and bone tissue, remarkably altered by 22 week ovariectomy. Moreover, all treatments significantly reduce the TNF-α mRNA expression in tibiae, and this cytokine, as well as IL-1 and PGE_2_, has been identified as regulator for osteoclast activity [35]. TNF-α presents anti-apoptotic activity on osteoclasts, increasing their lifespan [36] and synergistically with RANKL stimulates osteoclastogenic activity [37], inducing osteoclast formation and bone resorption both directly and increasing the sensitivity of maturing osteoclasts to RANKL. TNF-activated pathways and mechanisms involved in bone remodeling remain partially unclear; in fact, a recent review summarized the paradoxical role of TNF-α on bone homeostasis and bone remodeling imbalance involved in systemic/vertebral osteoporosis [38]. Other studies will be necessary to explain the lack of synergistic effect between SW and RAL on TNF-α in bone. A possible competitive effect between the two treatments, by unidentified endogenous endocrine or paracrine factors on bone, can not be excluded and should be clarified.

On the other hand, the association SW+RAL significantly reduces cathepsin k levels, a prominent lysosomal cysteine protease that is involved in bone degradation of extracellular matrix proteins, such as elastin and collagen [39]. Few years ago, it was demonstrated that two cathepsin K inhibitors prevented bone loss with similar efficacy to that of alendronate in estrogen-deficient rabbits, but, unlike bisphosphonates, had no suppressive effect on bone-formation rate at trabecular and cortical bone sites suggesting better long term benefits than the current standard of cure [40]. To date, odanacatib and ONO-5334 are currently in clinical development, as new reliable strategy for osteoporosis treatment [41].

Approximately thirty Bmps members of transforming growth factor-β superfamily have been identified and characterized, among which Bmp2 is recognized as a pivotal signal in regulating osteoblastogenesis [42]. Several studies using transgenic mice have shown that Bmp2 functions as a fundamental component of the inherent regenerative capacity of bone [43]. Bmps are used locally for the treatment of non-union fractures and proteosome inhibitors enhancing Bmp2 expression may have an effect on bone tissue and have been proposed as potential anabolic therapies [8]. The involvement of NO pathway in SW osteogenic effect was previously reported *in vitro* [44], evidencing an increase in Bmp2 and RUNX2 transcription in marrow stromal cells of hips with osteonecrosis. Here, we evidenced that SW and their association with RAL increase mRNA Bmp2, indicating their effects on bone regeneration, compromised in OVX rats. This data was strengthened by the significant increase in serum iPTH level induced by SW plus RAL, supporting the stimulatory effect on bone formation of the combined therapy.

During bone remodeling, beyond the resorptive role of osteoclasts, new bone is formed by osteoblasts derived from mesenchymal stem cells [45]. These latter ones could also differentiate into adipose cells, as it can occur during bone loss through an expansion of adipose tissue in the bone marrow [46]. Among factors having contrary effects on both differentiation pathways,RUNX2 is master transcription factor for osteogenesis [47], while PPARγ is designated to induce adipogenesis [48]. Interestingly, SW and RAL not only markedly increased the transcription of RUNX2 in bone, but also reduced PPARγ levels in bone marrow, indicating the shift of MSCs from adipogenesis to osteogenesis. This hypothesis was confirmed by the reduction of adiponectin in bone marrow from SW and RAL treated animals. In fact, clinical studies showed that high levels of adiponectin are associated with a reduction of bone mineral content and body mineral density [49, 50], that also confirmed in mouse models [51]. Clinical postmenopausal estrogen insufficiency was found to be associated with increased bone marrow adiposity [30], while the observed increase in marrow adipogenesis could be prevented or reversed by estrogen replacement both in humans and rats [29]. Our data confirm previous results showing that adipogenesis induced by OVX is a reversible process which can be corrected by estrogen, phytoestrogens, or raloxifene treatments [52], and for the first time we demonstrate the reduction of adipogenesis in rat bone marrow after repeated SW application. Interestingly, the lack of the synergic or additive effects by SW and RAL on adipogenesis/osteogenesis balance could represent a limit of their interplay; indeed, a possible competitive effect by unidentified endogenous endocrine or paracrine factors involved in their respective mechanism of action at bone marrow level can be considered.

In conclusion, our study demonstrates the beneficial anti-osteoporotic effects of SW therapy, alone or in combination with raloxifene, evidencing their strengths and limits. The mechanisms of these effects can be a result of the increase of bone formation and the reduction of bone resorption. Combined or multi-target therapies are a common approach in the treatment of chronic and multifactorial diseases, such as osteoporosis. SW therapy is considered a non-invasive therapeutic modality with effectiveness, convenience, and safety; also replacing surgery with no surgical risks in many orthopedic disorders [20, 53] and it may represent an innovative strategy to limit the progression of osteoporosis.
